# Draft Genome Sequence of the Bacterial Endophyte *Priestia megaterium* B1, Isolated from Roots of Apple (*Malus domestica*)

**DOI:** 10.1128/mra.01172-22

**Published:** 2023-05-18

**Authors:** Fatma M. Mahmoud, Souvik Kusari, Susanne Kublik, Sarah Benning, Roberto Siani, Sebastian Zühlke, Viviane Radl, Felix Mahnkopp-Dirks, Michael Schloter

**Affiliations:** a Research Unit for Comparative Microbiome Analysis, Helmholtz Munich, German Research Center for Environmental Health, Neuherberg, Germany; b Chair for Soil Science, Technical University of Munich, Freising, Germany; c Faculty of Chemistry and Chemical Biology, Center for Mass Spectrometry (CMS), TU Dortmund University, Dortmund, Germany; d Botany and Microbiology Department, Faculty of Science, Suez Canal University, Ismailia, Egypt; e Institute of Horticultural Production Systems, Section Woody Plant and Propagation Physiology, Leibniz University Hannover, Hanover, Germany; University of Maryland School of Medicine

## Abstract

Over the past years, a number of important traits supporting plant growth have been shown for different strains of Priestia megaterium (formerly known as Bacillus megaterium). Here, we report the draft genome sequence of the endophytic bacterial strain *Priestia megaterium* B1, which was isolated from surface-sterilized roots of apple plants.

## ANNOUNCEMENT

Priestia megaterium (1) is known for its plant-growth-promoting potential (2) and its ability to enhance plant resistance against stress (3, 4). *Priestia megaterium* strain B1 was isolated from surface-sterilized roots of a single apple plant and grown for 8 weeks in gamma-irradiated soil in Ellerhoop (Germany; latitude, 53.71435; longitude, 9.770143). Roots were surface sterilized by sequential immersion in 70% ethanol, 1.3 M sodium hypochlorite, and 70% ethanol. Crushed roots were plated onto water agar (WA; Difco, NJ) and incubated at 28 ± 2°C for 7 days. Isolates were purified on nutrient agar (HiMedia, Mumbai, India) and preserved in 15% (vol/vol) glycerol at −80°C (5).

Genomic DNA was extracted using the Genomic-tip 20/G kit (Qiagen, Hilden, Germany), after the cultivation of strain B1 from a glycerol stock in LB broth (Roth, Karlsruhe, Germany) for 48 h at 30°C. Multiplexed libraries were prepared following the protocol “Procedure & Checklist-Preparing Multiplexed Microbial Libraries Using SMRTbell Express Template Prep kit 2.0.” (Pacific Biosciences, CA). Genomic DNA was sheared to approximately 10 kb using g-TUBEs (Covaris, MA) followed by a cleanup step of the DNA using AMPure beads (Pacific Biosciences). To avoid the possible loss of plasmids, no size selection was included. The library was loaded by diffusion loading on a Sequel system (Pacific Biosciences). The loading concentration on the single-molecule real-time (SMRT) cell 1M v3 was 6 pM. Sequencing run parameters were 10-h movie time, 2-h immobilization time, and 2-h pre-extension time using the continuous long read mode.

Reads were obtained in FASTQ format, applying SAMtools (v1.12) (6). LongQC 1.2.0c (7) revealed an acceptable read quality, and thus, no error correction was performed. Flye v2.9 (https://github.com/fenderglass/Flye) was used for the *de novo* assembly of reads, using the command flye –pacbio-raw –threads –out-dir –scaffold (8). No circularization or gap closure was performed after the assembly. CheckM v1.0.18 (9) was used to calculate completeness and contamination percentages. Assembly quality was checked by QUAST v4.4 (10). Type (Strain) Genome Server (https://tygs.dsmz.de/) was used to calculate digital DNA-DNA hybridization (dDDH) values (11). Average nucleotide identity (ANI) was estimated by FastANI v0.1.3 (12). The genome was annotated using NCBI Prokaryotic Genome Annotation Pipeline (PGAP) v6.5 (13). Default parameters were used for all software.

Sequencing yielded a total of 426,737 reads (mean read length, 2,747.024 bp; *N*_50_, 3,979 bp). The resulting draft genome had a total sequence length of 5,664,514 bp (GC content, 37.93%; contig *N*_50_, 5,139,808 bp) comprising 13 contigs. The completeness of the genome was 99.43%, with 0.07% contamination. Both dDDH and ANI confirmed the identity of strain B1 as *Priestia megaterium*, with a nucleotide sequence homology of 73.3% and ≥95%, respectively. PGAP identified 5,710 coding DNA sequences, which included potential genes for siderophores production, phosphate solubilization, and resistance to heavy metals.

### Data availability.

The draft genome sequence was deposited at DDBJ/ENA/GenBank under the accession JARQZM000000000, via BioProject PRJNA700828 (with BioSample accession number SAMN30014917, SRA accession number SRX16711599, and GenBank assembly accession GCA_024582855.2).
